# Real World Efficacy and Safety Results of Ixazomib Lenalidomide and Dexamethasone Combination in Relapsed/Refractory Multiple Myeloma: Data Collected from the Hungarian Ixazomib Named Patient Program

**DOI:** 10.1007/s12253-019-00607-2

**Published:** 2019-02-02

**Authors:** Gergely Varga, Zsolt Nagy, Judit Demeter, Szabolcs Kosztolányi, Árpád Szomor, Hussain Alizadeh, Beáta Deák, Tamás Schneider, Márk Plander, Tamás Szendrei, László Váróczy, Árpád Illés, Árpád Bátai, Mónika Pető, Gábor Mikala

**Affiliations:** 1 3rd Department of Internal Medicine, Semmelweis University, H-1125, Kútvölgyi út 4, Budapest, Hungary; 2 1st Department of Internal Medicine, Semmelweis University, Budapest, Hungary; 3 1st Department of Internal Medicine, University of Pécs, Pécs, Hungary; 4 National Institute of Oncology, Budapest, Hungary; 5 Markusovszky University Teaching Hospital, Szombathely, Hungary; 6 Department of Hematology, Institute for Medicine, Clinical Center, University of Debrecen, Debrecen, Hungary; 7 Department of Hematology and Stem Cell Transplantation, South Pest Central Hospital, National Institute for Hematology and Infectious Diseases, Budapest, Hungary

**Keywords:** Multiple myeloma, Relapsed, Ixazomib, Named patient program

## Abstract

Ixazomib-Revlimid-Dexamethasone is an all-oral treatment protocol for multiple myeloma with a manageable tolerability profile which was available through a named patient program for Hungarian patients from December 2015 to April 2017. We analyzed the clinical characteristics and survival of 77 patients treated at 7 centers within this program. The majority of patients responded, we found complete response in 9, very good partial response in 8, partial response in 32, minor response or stable disease in 13 and progressive disease in 11 patients. Progression free survival was 11.4 months. There was a trend of longer progression free survival in those with 1 vs. >1 prior treatment, with equally good effectivity in standard risk and high risk cytogenetic groups. The adverse events were usually mild, none leading to permanent drug interruptions. There were 5 fatalities: 3 infections and 2 pulmonary embolisms. Our real word data support the use of Ixazomib-Revlimid-Dexamethasone as a highly effective and well tolerated oral treatment protocol for relapsed myeloma.

## Introduction

It was 15 years ago that introduction of the intravenous proteasome inhibitor (PI) bortezomib revolutionized the treatment of multiple myeloma, soon followed by the approval of lenalidomide for the same disease. Ixazomib, however, is the first oral PI that showed high efficacy and safety for the treatment of relapsed and/or refractory multiple myeloma in the TOURMALINE MM1 trial in combination with lenalidomide and dexamethasone (IRD) leading to its FDA and subsequent EMA approval. Its indication stands as: relapsed/refractory multiple myeloma (MM) that have received at least one prior therapy [1]. IRD is an all-oral treatment protocol with a manageable tolerability profile in patients with MM including those with high cytogenetic risk [2, 3].

Nevertheless, clinical trials are significantly different from real world use of therapies in many aspects including less rigorous patient selection, greater flexibility with dosing and country specific funding restrictions. This makes reporting of real world data very important for clinicians. Regarding the IRD triplet combination, available real world data are currently very limited [4–6].

Following its FDA approval, from December 2015 to April 2017 IRD treatment was available for Hungarian patients with relapsed MM treated with 1–3 prior lines of therapy through a Named Patient Program (NPP) by Takeda Parmaceuticals. Inclusion criteria followed that of the pivotal trial [1], and are summarized in Table 1. Protocol treatment continued until progression or death; several patients are still taking the medications.

**Table 1:** Eligibility criteria of the ixazomib named patient program

Patient is = > 18 years of age	
Patient is diagnosed with multiple myeloma according to standard criteria	
Patient has received 1 to 3 prior lines of therapy	
Patient is in biochemical or symptomatic relapse and is not on an active anti-myeloma therapy (except for steroids) at the time of this application	
Next planned therapy for the patient is ixazomib in combination with lenalidomide and dexamethasone	
Patient is not refractory to lenalidomide or a proteasome inhibitor, or was not refractory to lenalidomide or proteasome inhibitor-based therapy at any line.	
Absolute neutrophil count = > 1000/mm3 and platelet count = > 75,000/mm3;	
Total bilirubin <= 1.5 x upper limit of normal;	
Alanine aminotransferase and aspartate aminotransferase <= 3 x upper limit of normal;	
Calculated creatinine clearance = > 30 mL/min	
Patient has an ECOG performance status score of 0, 1 or 2	
Both males and females have agreed to use an effective contraception method during and for 90 days following treatment.	

## Aims and Methods

The aim of this retrospective study was to evaluate the efficacy and safety of IRD in the real world practice in a retrospective analysis using case records of patients taking part in the Hungarian Ixazomib NPP. Its scope was to analyze international staging system (ISS) and fluorescent in situ hybridization (FISH) status, response rate, progression free survival (PFS), treatment duration, adverse events (AEs), as well as dose modifications and treatment discontinuations.

Patients who have not completed at least one full cycle were excluded. Patients were continued to be followed up if treatment was stopped due to side effects, administrative or regulatory reasons, but were censored with that date if a new therapeutic line was started without fulfilling the IMWG criteria for progressive disease. Response criteria (complete response [CR], very good partial response [VGPR], partial response [PR], no response [NR], and progressive disease [PD]) and survival measures (progression-free survival [PFS] and overall survival [OS]) were defined according to published International Myeloma Working Group guidelines [7]. Statistical analyses were performed using the SPSS (version 20.0) software package. The way how FISH testing was performed varied with no plasma cell selection in the majority of the centers. There was no consensus regarding the probes used but those for 17p deletion, translocations (11;14), (4;14) and (14;16) and 1q amplification were generally part of the set. FISH results were available in 54 out of the 77 patient. For the purpose of this study, patients with t(4;14), t(14;16), 1q amplification and del(17p) were grouped together as a high risk cohort.

The study was approved by the Hungarian National Ethics Committee, the patients provided full informed consent, and treatment inclusion of each and every patient was individually approved by the Hungarian National Institute of Pharmacy.

## Results

### Patient Characteristics

77 patients entered the program and were treated at 7 centers, their clinical characteristics are presented in Table 2. Three cases were excluded from the efficacy analyses as they had not completed a single full cycle of therapy. Patients were younger than the usual myeloma patient at this stage (median age was 66 years), and were heavily pretreated. The median line of prior treatment was 2, the percentage of patients having had 1, 2 and 3 prior cycles were 29, 34 and 37%, respectively. Except for one patient, all had both IMiD and PI pretreatment in various combinations depending on the era when it was initiated. In some patients prior treatment lines spanned over several years, others were refractory and therefore exhausted their options quickly.

**Table 2:** Patient characteristics prior to start of the IRD treatment. This table includes 77 patients, however 3 of them who did not complete the first course were excluded from the efficacy analyses

	n (%)
All cases	77
Had <1 cycles of IRD	3 (3.8%)
Sex (M/F)	42 (54%)/35 (46%)
Number of prior lines (1, 2, 3)	21 (27%), 27 (35%), 30 (39%)
Prior Bortezomib	76 (99%)
Thalidomide	77 (99%)
Transplantation	45 (58%)
Refractory to last line	2 (2.6%)
ISS 1	28 (36%)
2	9 (11%)
3	17 (22%)
not performed	23 (30%)
FISH high risk	21 (27%)
standard risk	33 (43%)
not performed	23 (30%)
Mean number of IRD cycles	8.3
Ixazomib dose reduction required	9 (11%)
Permanent interruption due to AEs	5 (6.5%)

The availability of lenalidomide at the time of this program was limited, and this affected the number of potential patients. Lenalidomide was only funded through an individual patient funding scheme which required 3 monthly renewals that were not automatically granted. NPP applications were filed usually for patients who have run out of other options, or had side effects limiting the reuse of thalidomide or bortezomib. This explains why the number of prior lines is higher in this cohort compared to others. Failure to secure lenalidomide access for the subsequent cycles has led to protocol interruption in some cases.

### Adverse Events

According to the reported number of AEs (Table 3) IRD treatment was well tolerated, AEs above grade 1–2 were rare. The most common AEs were hematologic toxicities (mostly thrombocytopenia) and infections. Five fatalities were recorded, 3 infections (2 pneumonias and 1 neutropenic sepsis) and 2 thrombotic events (both of them were pulmonary embolisms, as prophylaxis for one had aspirin, the other low molecular weight heparin but was non-compliant).

**Table 3:** Adverse events

	Grade 1–2 n (%)	Grade 3 n (%)	Grade 4 n (%)	Grade 5 n (%)
Neutropenia	7 (9.1%)	6 (7.8%)		
Anemia	2 (2.7%)	4 (5.2%)		
Thrombocytopenia	8 (10.3%)	4 (5.2%)	2 (2.6%)	
Infection	10 (12.9%)	4 (5.2%)		3 (3.8%)
Diarrhea	7 (9.1%)	1 (1.3%)		
Skin rash	2 (2.7%)			
Thromboembolism				2 (2.7%)
Cardiology	1 (1.3%)	1 (1.3%)		
All	37 (48.1%)	22 (28.6%)		5 (6.5%)

There were no permanent drug interruptions due to AEs, except the 5 fatal cases. In 3 patients, however, IRD triplet treatment was stopped prematurely due to logistic reasons: 2 patients (1 in PR and 1 in VGPR) stopped ixazomib and carried on lenalidomide maintenance only, and the third with stable disease switched to a new protocol.

Ixazomib dose reduction from 4 to 3 mg weekly dose happened in 9 patients, who then could continue on the protocol. Reasons were mostly thrombocytopenia and diarrhea. Further dose reduction to 2.3 mg was only necessary in one case. Data regarding lenalidomide dose reductions and colony stimulating factor use were not collected, but these were both utilized in every participating center, according to standards of care.

### Efficacy

**Fig. 1:**
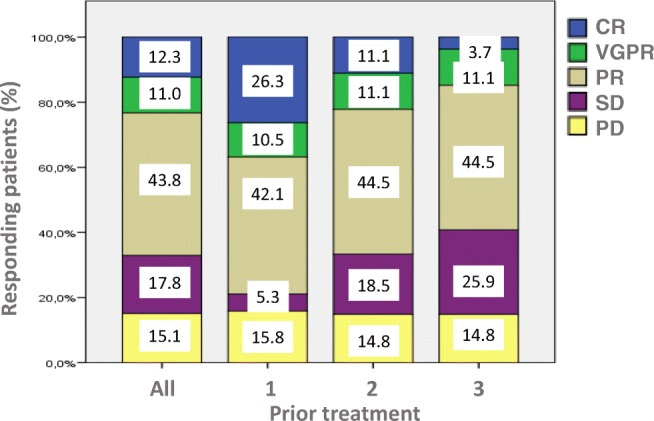
Response rate according to the number of prior treatments. There are numerically more CRs in patients with less prior treatment, but the differences are not significant

Among the 74 patients who completed at least one cycle of treatment, IRD was effective in the majority of patients. The best response was CR in 9, VGPR in 8, PR in 32, MR or SD in 13 and PD occurred in 11 patients, one patient had no formal disease re-assessment. Response according to ISS, FISH and number of prior lines of treatment is presented on Fig. 1.

**Fig. 2:**
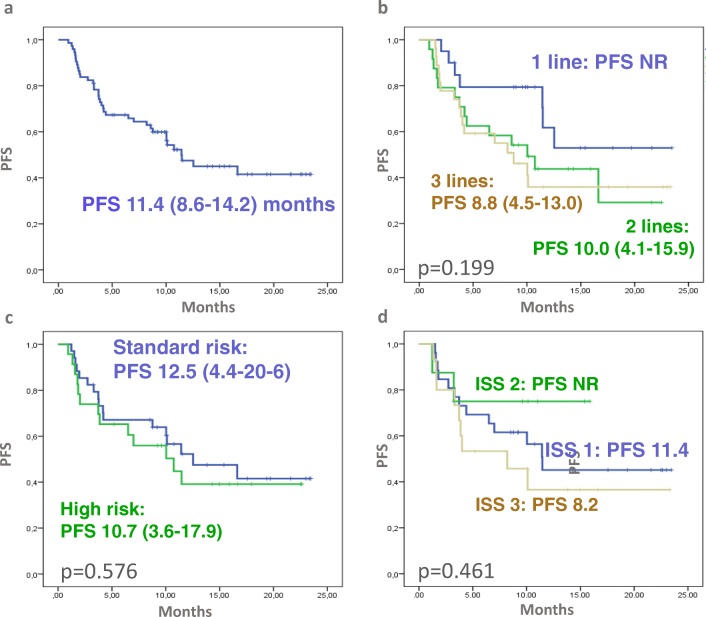
Progression free survival (PFS) of NPP patients. (**a)** PFS of all patients, (**b**) PFS according to prior lines, (**c**) ISS, (**d**) FISH risk status. There was a trend of longer PFS in those with 1 vs >1 prior treatment (*p* = 0.074). Importantly there was no significant difference between standard risk and high risk FISH and ISS patients

After 12.5 months median follow up and 39% of patients still on treatment, the calculated PFS was 11.4 months (Fig. 2a). There was a trend of longer PFS in those with 1 vs. >1 prior treatment (*p* = 0.074, Fig. 2b), but there was no significant difference between standard and high risk FISH patients and the 3 ISS categories (Fig. C, D). However, the 3 cases where deletion 17p was demonstrated at the time of relapse (clonal evolution) fared very poorly. Similarly, in cases of clinically aggressive disease, such as plasma cell leukemia (PCL) and extramedullary disease (EMD) IRD was less effective (data not shown).

## Discussion

There are plenty of differences between the Hungarian cohort and the published TOURMALINE MM1 trial data highlighting some of the typical differences between a highly selected trial population and real life patients [1].

A recent review merged the Czech (*n* = 57) the British (*n* = 46) and the Greek (*n* = 35) NPP data, and published their results (*n* = 138) [4]. They also excluded patients who have not completed at least one cycle. In this group, the median age was 68 years compared to 66 in our patients, M/F ratio was 85/53, with ECOG 0–1: 71.8% and ECOG 2–3: 27.2% of patients. The median number of previous therapies was 1.5 (range: 1–7) which is less than in our group (median 2). In this group 50.0% of patients had received one line of prior treatment, 30.4% two lines, 14.5% three lines and 5.1% had received 4–7 prior lines of anti-myeloma therapies. The PI pre-treatment was very frequent (94.2%) however only 59.4% of these patients had prior IMiD, and 26.1% had autologous transplantation (ASCT). Our group was more heavily pretreated, with virtually all patients having had both PI and IMiD, and 58% had prior ASCT.

The explanation for the significantly higher number of previous treatments with the Hungarian NPP is clear: at the time of this program the health authorities would not have funded lenalidomide without prior utilization of both thalidomide and bortezomib and also required the demonstration of a reason why retreatment with one of them was not feasible. As a result lenalidomide was rarely used at first relapse at the time when this program began.

Patient-reported health-related quality of life was a secondary endpoint of TOURMALINE trial, which demonstrated that addition of ixazomib to lenalidomide-dexamethasone (Rd) significantly improved efficacy while quality of life was maintained [8]. Also exposure-adjusted rates of hospitalization were similar between the ixazomib-Rd and placebo-Rd arms [9, 10].

In the Czech-UK-Greek cohort the median follow-up was 9.1 months, and the median treatment duration 7.2 months, which is similar to our findings. 10.8% of patients reached sCR/CR, 18.5% VGPR, 39.2% PR, 4,6% MR, 10.0% SD and 16.9% PD, which is very similar to what we have found. The ORR (≥PR) was estimated at 68.5% in the overall population; 76.6% in patients receiving IRD as second line, 64.3% in those as third line and 55% in those beyond third line, again similar to the results of the Hungarian cohort (78.9, 66.6, 59.2 in the same groups).

Similarly to our findings, treatment discontinuation rate was low (13.7%) in the Czech-UK-Greek cohort. Interestingly, 28.3% of their patients experienced peripheral neuropathy, while in our study significant worsening or newly developing neuropathy was not reported during IRD. Data regarding preexisting neuropathy was not collected.

In this study by Terpos and coworkers, the estimated median PFS was 27.6 months that is significantly better than what we found, but also better than the response level the pivotal TOURMALINE study demonstrated (20.6 months). In our patients with 12.5 months median follow up and 39% of patients still on treatment, the calculated PFS was approximately 1 year (11.4 months). The PFS, however, of those patients who reached at least stable disease was 16.6 months. Importantly, the effect of the known prognostic markers (FISH, ISS) was not significantly influencing the PFS, although, patients with high risk clinical features (plasma cell leukemia, extramedullary disease) had poor outcome. In this regard our data support the TOURMALINE study results that showed equally good effectivity in LR and HR cytogenetic groups. This finding is probably related to the prolonged PI exposure that is not easy to deliver with parenteral drugs. There was a trend of longer PFS in patients with only one prior line of treatment compared to those with two or three lines.

Probably as a consequence of the more prior treatment lines, the proportion of high risk patients were enriched in the Hungarian cohort. Terpos et al. did not report ISS and FISH findings, but if our patient cohort is compared to the TOURMALINE trial patients, we can demonstrate significantly more ISS 3 and HR FISH patients in our group [2, 11].

Why did we enter a lot of high risk patients into this program? On the one hand, at the time of the NPP daratumumab and carfilzomib were not available in Hungary and ixazomib was an attractive third agent to add to lenalidomide especially as it demonstrated good efficacy in HR patients in the TOURMALINE trial in which many of the Hungarian centers actually participated [11].

Additionally, entering to the program was very quick through online registration, therefore rapid disease progression was not an obstacle. As opposed to this, in clinical trials due to the many requirements needed to fulfill during screening and the required drug washout periods, patients can be excluded from enrollment when there is an urgent need to start effective treatment.

These reasons probably explain the shorter PFS of the Hungarian NPP cohort when compared to the Czech-UK-Greek NPP cohort and the TOURMALINE data. In our dataset less than a third of patients were treated at first relapse, whereas in the TOURMALINE trial the majority were included in this clinical situation. Additionally, in the TOURMALINE trial only 21% had high risk cytogenetics which was present in 38% in our patient cohort. This fact probably accounts for the shorter PFS observed in our more heavily pretreated patient group.

We conclude that IRD proved to be a safe and effective treatment option for real-life relapsed/refractory MM patients in our country. Compared to the TOURMALINE study results, the PFS of our cohort is somewhat inferior that is not unusual when real life patient data are compared to randomized controlled study results. Our NPP results further highlight the differences between patients treated in routine practice and those treated in clinical trials, however, fully confirm the safety and efficacy of the ixazomib-lenalidomide-dexamethasone all-oral combination in relapsed/refractory multiple myeloma. We believe that an all-oral salvage regimen such as IRD remains a valid option for the treatment of MM patients even in light of possibly newer and more effective but parenteral drug combinations [12] as this combination requires less frequent outpatient visits and allows patients to maintain their usual lifestyle.
